# Stakeholder Insights from Zika Virus Infections in Houston, Texas, USA, 2016–2017

**DOI:** 10.3201/eid2411.172108

**Published:** 2018-11

**Authors:** Stephanie R. Morain, Catherine S. Eppes, Joslyn W. Fisher, Courtenay R. Bruce, Martha Rac, Kjersti M. Aagaard, Rebecca Lunstroth, Savitri Fedson, Pallavi Dinesh, Jean L. Raphael

**Affiliations:** Baylor College of Medicine, Houston, Texas, USA (S.R. Morain, C.S. Eppes, J.W. Fisher, C.R. Bruce, M. Rac, K.M. Aagaard, S. Fedson, P. Dinesh, J.L. Raphael);; McGovern Medical School at the University of Texas Health Science Center, Houston (R. Lunstroth)

**Keywords:** communicable diseases, emerging, Zika virus infection, electronic health records, health information technology, health policy, Houston, Texas, United States, viruses, Zika, Zika virus

## Abstract

Responding to Zika virus infections in Houston, Texas, USA, presented numerous challenges across the health system. As the nation’s fourth-largest city, in a subtropical region with high travel volume to Latin America and the Caribbean, Houston was an ideal location for studying experiences encountered by clinicians and public health officials as they responded to the Zika virus crisis. To identify the challenges encountered in the response and to explore strategies to improve future responses to emerging infectious diseases, we interviewed 38 key stakeholders who were clinical, scientific, operational, and public health leaders. From the responses, we identified 4 key challenges: testing, travel screening, patient demographics and immigration status, and insufficient collaboration (between public health officials and clinicians and among clinical providers). We also identified 5 strategic areas as potential solutions: improved electronic health record support, specialty centers and referral systems, standardized forms, centralized testing databases, and joint academic/public health task forces.

In February 2016, the World Health Organization (WHO) declared the cluster of cases of microcephaly and other neurologic abnormalities associated with Zika virus a public health emergency of international concern. Since 2015, this virus has infected >1 million persons in 70 countries (*1*). From 2015 through December 2017 in the United States and its territories, >42,000 laboratory-confirmed symptomatic cases were reported and ≈7,000 pregnant women had laboratory evidence of possible Zika virus infection (*2**,**3*).

Responding to Zika virus presented numerous challenges across the health system. Zika virus research before 2015 was scarce, leaving clinicians and public health policy makers with little guidance regarding the virus’s natural history, rate of perinatal transmission, or mechanisms or rate by which infections triggered microcephaly and other severe congenital abnormalities. Furthermore, diagnostic tools were limited because of Zika virus cross-reactivity with other flaviviruses on serologic assays, complicating individual diagnoses and population-based serosurveillance (*4*).

To develop guidelines to support the clinical and public health response, the Centers for Disease Control and Prevention (CDC) mobilized rapidly. Nevertheless, considerable work was needed by those on the ground to translate CDC guidelines and other emerging research into actionable policies at the institutional level.

The challenges encountered by clinicians and public health officials when responding to the emerging Zika virus crisis in Houston, Texas, USA, from January 2016 through June 2017 were ideal for a case study. As the nation’s fourth most populous city and with >10 million annual international travelers, Houston is a global gateway to Latin America and the Caribbean, putting it at high risk for travel-associated cases (*5*). Furthermore, the city’s subtropical bayou setting enhances the threat of locally acquired transmission from *Aedes aegypti* and *Ae. albopictus* mosquitoes (*6*). During 2015–2017, a total of 365 Zika virus cases, including at least 7 transmitted by local mosquitoes, were reported in Texas (*7*). In a 7-month period, 105 pregnant patients were referred to a specialty clinic for potential Zika virus exposure; 75 met testing criteria and 8 ultimately had positive test results, a screen-positive rate of 11% (*8*).

To explore the clinical and public health responses to Zika virus in Houston, we interviewed expert stakeholders. We report the key challenges they encountered and propose strategies to inform the response to Zika virus and future emerging infectious diseases.

## Methods

We conducted semistructured interviews of 38 clinical, scientific, and public health experts in Houston and current or former Texas public health officials (Table 1). Almost half (45%) worked in the fields of obstetrics or pediatrics, and the majority of clinicians were affiliated with an academic medical center. The interview guide elicited participants’ perceived challenges related to Zika virus infection prevention, testing, and clinical management, as well as strategies for addressing those challenges. During April–June 2017, a researcher with doctoral training in qualitative methods (S.R.M.) conducted the interviews. When possible, interviews were conducted in person (n = 24); the others were conducted via telephone. All interviews were audiorecorded, transcribed, and reviewed for accuracy. We analyzed transcripts by using MAXQDA (https://www.maxqda.com) and used qualitative coding to identify key themes related to challenges and strategies in the Zika virus response.

**Table 1:** Roles of key stakeholders interviewed with regard to Zika virus infections in Houston, Texas, USA, 2016–2017

Primary role	No. respondents
Pediatrics/neonatology	9
Obstetrics–gynecology or maternal–fetal medicine	8
Public health	8
Pathology	6
Infectious disease	3
Operations/leadership	2
Nursing	1
Genetic counseling	1
Total	38

## Challenges

From stakeholders’ discussions of the challenges encountered in responding to Zika virus infection, 4 primary themes emerged. These themes were testing, travel screening, patient demographics and immigration status, and collaboration (between public health entities and clinicians and among clinical providers) (Table 2).

**Table 2:** Challenges expressed by key stakeholders with regard to Zika virus infections in Houston, Texas, USA, 2016–2017*

Challenge	Quotation
Testing	
Logistical burdens with collecting and submitting samples	“I was filling out a form for the city. I was filling out another form for the state, and another for CDC. All to just be able to submit the samples for testing… it took me about 15–20 minutes just to fill out the paperwork [per patient]. And a lot of it was redundant.”—infectious disease specialist
Delays in receiving laboratory results	“… for a lot of women, [test results are] going to make no difference at all because they are going to continue their pregnancy... but, for other women, it may completely change their decision-making…. So that turnaround time matters, absolutely.”—maternal–fetal medicine specialist
Complexity and limitations of available Zika virus tests	“The testing is not very specific. It doesn’t necessarily eliminate your risk of having Zika, so there’s lots of limitations even with a negative test.”—academic pediatrician
Influence of commercial testing	“Frankly, the commercial labs–they’re a blessing and not so much a blessing at the same time… when PCR specimens are done in a commercial lab and they’re positive… we may have a patient name and that’s it. Maybe their age, maybe their address, maybe not. And so we don’t have all of the demographic information and epidemiologic information that we’d like to have to do a full case investigation.”—state official
Poor mechanisms for exchanging laboratory data	“We get [Zika test results from the health department] through the fax… and we’ll have medical records scan it in and then I sent that to the provider who is seeing the patient. It’s a little clunky, but that’s the only way we can do it because of the mode that we’re getting it through the fax.”—community obstetrician
Travel screening	
Insufficient clinician initiation	“We would love it if our safety net providers… were doing a similar type of Zika screening for all patient visits, not just OB visits, ‘cause you’re kind of behind the ball if you wait ‘til the person’s already pregnant and has been exposed.”—public health physician
Inaccurate referral information	“So I think particularly for the immigrant population here in Harris County, there is also concerns that, ‘why are they asking those questions, do they want to know where I’ve been and what I’ve done?’ So I think there is also the concern for people who are here illegally perhaps that they don’t want to divulge their travel history.”—maternal–fetal medicine specialist
Insufficiently precise information	“… pathology would receive a blood sample on a mom who had been to Florida. She said yes to Florida… but based on the form that pathology got, it doesn’t say the city that she visited. Before they will send it, they have to verify that it was Miami. I call the mom, well, she went to Jacksonville. She didn’t go to Miami. That kind of stuff is very time intensive for somebody to follow up on.”—genetic counselor
Patient demographic and immigration status	
Transient and low socioeconomic level population	“… A lot of these patients are very underprivileged and have very low resources, living in charity homes, living in homeless shelters…. How do we provide resources for these patients that have almost no resources to begin with? ... that’s a big issue that I’m not really sure how to fully tackle. I think it’s a very large issue”—academic pediatrician
Language barriers	“… 100% of our moms were Hispanic and low income. I can’t remember a single one of them that spoke English either. And so there’s a dynamic of we’re trying to have interviews with them in a language that a number of our epidemiologists don’t speak and try to find translators to convey whatever we’re trying to ask, but then there’s the dynamic of these patients with their own providers… there’s a loss of information there just on the basis of translation.”—public health physician
Undocumented immigration status	“… we’re definitely hearing from some people… parents who are not here legally—even if their kids are here legally—are afraid to access medical care for fear of deportation.”—community pediatrician
Collaboration among public health clinicians	
Confusion as to appropriate Zika virus “point person” within public health system	“We [academic medical centers] are the laboratories that are actually going to see those patients come in with [an infectious disease] … when you have these brand-new, emergent infections… that line of communication is not well-established. Who’s in charge of that at public health? We don’t always know.”—academic pathologist
Poor communication of testing results to patients	“‘They [the public health epidemiologists] say things like, ‘You don’t have anything to worry about, your IgM is negative.’ What they don’t know is that this patient’s been persistently viremic… and we were very concerned and in fact that patient had an affected fetus… and so then we have to call the Health Department and say, ‘Yeah, they have a positive PCR.’”—maternal–fetal medicine specialist
Collaboration among clinicians	
Poor communication between obstetrics and pediatric teams	“… the joke always goes as pediatricians, we knew where babies came from but we didn’t know how they got there.... I don’t think there’s a highly reliable system across the state that ensures that OB providers are giving appropriate information to the [pediatric] team.”—public health official
Questions of case “ownership”	“Follow-up for our babies was a big [issue] … who was actually going to do the follow-up? ... They’ve passed or they’ve failed their hearing test, so now where do we send them? ... it was just a big black hole.”—academic pediatrician

*CDC, Centers for Disease Control and Prevention; OB, obstetrics; Pedi, pediatrics.

### Testing

The most commonly described challenges were associated with Zika virus testing. Every clinician interviewed described such challenges. Five testing issues emerged.

First, clinicians described logistics burdens associated with collecting and submitting samples to public health departments, characterizing the paperwork and approval processes as “redundant,” “very time-consuming,” and “a significant barrier to care.” One case described by an obstetrician exemplifies these challenges. To order serologic testing, the obstetrician had to complete hospital send-out laboratory forms and 3 forms from the Houston Health Department (HHD) and telephone HHD for approval. When the test results returned positive, the clinical team sent a plaque-reduction neutralization test (PRNT) sample to CDC to rule out cross-reactivity with other flaviviruses (*9*). While awaiting PRNT results, the patient elected amniocentesis, which required completion of 5 more forms and separate HHD approval. At the time she gave birth, the PRNT results were still pending, so the clinical team submitted placental samples for testing, which required completion of 4 new forms and involvement of the state health department.

Second, clinicians expressed concerns regarding the clinical effects of delayed receipt of test results. These delays were generally longest early in the response, before testing was available via in-house or commercial laboratories and as public health departments faced extensive delays in federal funding to support testing. The delays were particularly challenging given Zika virus’s potential effects on fetal development and the relatively short duration of pregnancy. Test results could influence decisions about clinical management; the risks and benefits of amniocentesis or other testing; and reproductive decision-making, including pregnancy termination. As one maternal–fetal medicine specialist summarized, “It’s very difficult to base your management decisions on a test that took 6 to 8 weeks in pregnancy.”

The third challenge was the complexity and limitations of existing tests, including cross-reactivity with other flaviviruses and the contemporary general understanding of a limited period for IgM detection, which complicated determination of exposure and risks to pregnancy. It was believed that although IgM is expressed as early as several days after exposure, it typically wanes within 3 months, creating challenges for patients with a long duration of exposure or case testing delays of several months. These limitations frustrated efforts to identify true positive exposures and presented challenges for patient education. To quote a maternal–fetal medicine specialist, “The testing is not very specific… it doesn’t necessarily eliminate your risk of having Zika… that has been difficult to get our patients to understand. Because most of our patients think that if a test is negative, then the risk is eliminated.”

Fourth, several respondents noted the effects of commercially developed tests. According to respondents, commercial tests generally improved result turnaround times, partially alleviating the demand on scarce public health department resources. However, they also introduced new cost pressures, particularly for public institutions, compared with free services available through public health laboratories. Commercial testing also introduced challenges for public health systems because these results often lacked necessary demographic and epidemiologic information to support downstream case investigations of positive test results by local health departments.

Fifth, respondents described poor mechanisms for sharing data between laboratories and providers, including insufficient or delayed electronic health record (EHR) integration for ordering and reporting test results. Clinicians reported resorting to “clunky” workarounds, such as receiving a facsimile from the health department that then had to be scanned into the medical record. Providers expressed concern that such systems could lead to insufficient follow-up, particularly for care of neonates. For example, a pediatrician cited the challenges of exchanging and recording testing data as the reason why one infant in her practice was not evaluated for congenital Zika syndrome until 4 months of age—months beyond the CDC recommendations for evaluation and management of possible congenital infection (*10*).

### Travel Screening

We identified 3 themes associated with travel screenings to identify patients with potential Zika virus exposure. First, specialty referral centers reported receiving inaccurate travel histories from patients or referring clinicians. In some circumstances, respondents cited the inaccuracies as probably stemming from patient concerns over divulging travel history because of their immigration status. One obstetrician offered the example of a patient who had become pregnant in Central America and subsequently traveled to the United States. When entering the United States, the patient’s husband was detained as an undocumented immigrant and remained incarcerated throughout her pregnancy. The patient subsequently declined to disclose travel history to healthcare providers in Zika virus–endemic regions during several prenatal visits, ultimately notifying providers only of her travel early in her third trimester. According to another respondent, high levels of Zika virus–related anxiety may have motivated some patients to misrepresent their travel history in an effort to be referred for diagnostic testing.

Second, clinicians suggested that insufficient initiation of travel screening probably resulted in some Zika virus–exposed patients “fall[ing] through the cracks” and not receiving recommended evaluation or testing. Some cited institutional barriers, including lack of development or implementation of travel screenings or restricting travel screening to obstetric visits only instead of expanding to primary care and family medicine.

Third, reported travel information was sometimes insufficient or imprecise. For example, a patient might report visiting “Florida” when there was considerable heterogeneity in risk within the state (e.g., in the summer of 2016, a trip to Miami would trigger testing whereas a trip to Jacksonville would not) (*11*) or might list only the month of travel when the exact dates are necessary for ascertaining the most appropriate testing method.

### Patient Demographics and Immigration Status

According to respondents, patient demographics presented challenges for access to care and subsequent follow-up. Many patients undergoing evaluation and subsequent care for Zika virus exposure were from or had close family ties to Zika virus–endemic countries. Several respondents characterized patients as often “transient” and described extensive socioeconomic barriers, including lack of stable housing, transportation, or telephone service—all of which could undermine long-term follow-up and care coordination. As a pediatrician explained, “We have a patient that gave us their cell phone number, but the cell phone went dead. They don’t have family in the country and they live in a shelter, so I’m just not sure how to ensure we have good communication, transportation, follow up, and shelter.” Furthermore, respondents noted that for many patients, proficiency in the English language was limited, which respondents described as potentially exacerbating not only the accuracy of travel screenings but also patient education about Zika virus prevention and clinical management.

Several respondents also described extensive issues related to immigration status and implications for healthcare access. Some patients were reluctant to disclose their travel history for fear of deportation. Respondents also noted that concern about immigration status may have dissuaded some patients from seeking care, either during the prenatal period or for subsequent follow-up infant care. Other clinicians noted that immigration status influenced patient behavior across the health system. For example, public health clinicians described a “marked decline” in women accessing services through the Women, Infants, and Children program, reportedly because of concerns over deportation.

### Collaboration

We identified 2 types of collaboration challenges. One was between providers and public health agencies and the other among clinicians involved in patient care.

#### Provider–Public Health Agency Collaboration

Respondents described challenges with communications between clinicians and public health agencies at the local, state, and federal levels. To develop an action plan, the Houston Office of Surveillance and Public Health Preparedness convened local stakeholders, including city departments, researchers, and industry leaders (*12*). Nevertheless, respondents reported missed opportunities for interdisciplinary communication. For example, some clinicians described uncertainty identifying the Zika virus “point person” or team within health departments who could field questions from clinicians and hospital laboratories about case identification and testing or even which health department had responsibility. Furthermore, submitting laboratory testing for one patient could involve numerous health departments, including separate paperwork, processes, and phone approvals for each department—all of which presented additional time burdens for clinicians and delays for patient diagnosis.

According to one public health respondent, such challenges may reflect misunderstandings associated with allocation of responsibility across different public health partners: “I think there’s a fundamental misunderstanding of what the CDC does and doesn’t do. They have national experts. They provide resources that they get from Congress. There’s a national laboratory, but they don’t take over a response. A response is formulated at the local level… [but] local health departments have varying levels of expertise.”

#### Interclinician Collaboration

Several clinicians described interclinician communication about patient care as the “most frustrating” or “hardest” aspect of the Zika virus response. We identified 2 distinct issues. The first and by far most common problem was insufficient or inefficient communication between obstetric and pediatric care providers, including pediatrician notification of suspected or confirmed maternal Zika virus infections. Specific criticisms related to this process included absence of a centralized database of positive test results, lack of connectivity between EHRs across different health systems, and an inability of pediatricians to access relevant maternal medical records.

Second, several clinicians described insufficient clarity regarding case “ownership.” Issues included disagreement over which provider or care team was responsible for tracking and communicating test results to patients and which providers (e.g., general pediatricians vs. specialists) should primarily be responsible for long-term evaluation of Zika virus–exposed infants.

## Strategies for Improvement

### Improved EHR Support

Respondents identified numerous ways in which technology could streamline testing processes, starting from the initial stage of screening patients, progressing to collecting and submitting samples, and then documenting and sharing results among providers and public health systems. Five specific respondent suggestions for improved EHR support were 1) standardizing screening questions within the patient’s EHR to ensure accurate assessment of risk exposure; 2) implementing EHR-based decision support systems to help providers select and order the appropriate test(s); 3) enabling electronic ordering of Zika virus testing through the EHR; 4) prepopulating demographic information within the test form to reduce provider time burden and improve the efficiency of public health case investigations; and 5) integrating the testing laboratory and patient EHR to enable test results to be automatically entered into the patient EHR.

Admittedly, revising an EHR system can be an unwieldy process (*13**,**14*). Institutions should anticipate this challenge and develop policies to enable timely integration of hospital EHRs to facilitate clinical care and public health reporting.

### Specialty Centers and Referral Systems

The rapid evolution of knowledge and the complexity of Zika virus testing and ongoing patient management provide arguments for specialty centers and referral systems. The education, documentation, and reporting requirements associated with patient care in the context of an evolving infectious disease outbreak are extensive and yet are only one of several goals during a patient–provider encounter. As one maternal fetal medicine specialist explained, “This is a pretty specialized area that’s rapidly evolving, and it’s probably good to send patients for at least a discussion with folks that really have their fingertips on what did we learn last night at midnight when The New England Journal [of Medicine] was released.” Several specialty centers were offered as corollaries, including existing models for perinatal HIV infection, congenital cytomegalovirus infection, or newborn screening for genetic conditions, as well as the designation of 3 tiers of health centers in the 2015 domestic Ebola response (*15*). For example, Illinois’s Perinatal Rapid Testing Implementation Initiative for HIV successfully reduced the number of mother–infant pairs who were discharged with unknown HIV status; the initiative created 4 regional networks, a 24-hour hotline, a surveillance system, and implementation resources including template policies and consent forms (*16*).

Specialty centers and referral systems offer several advantages for managing Zika virus infection and other emerging infectious diseases, including state of the science laboratory and ultrasonography/magnetic resonance imaging with high precision and efficiency, potential to scale up for pandemics and endemic-disease expansion, capacity for prenatal and postnatal diagnosis and postnatal follow-up, follow-up care, and opportunities for research and testing of potential interventions in an enriched population with a higher likelihood of exposure and infection.

Some patients, particularly undocumented immigrants or other patients of low socioeconomic status, may face financial and transportation barriers with regard to accessing specialty centers, particularly for repeated visits. Additional cost pressures may arise in association with imaging and specialty care, even among insured patients. Additional work is needed to identify strategies to improve access while managing patient and system costs; these strategies include using telemedicine, providing of transportation support and cell phones or phone cards to enable follow-up, and implementing of corresponding regulatory and payment structures to facilitate these services.

### Standardized Forms

Several clinicians suggested standardizing forms across local, state, and federal agencies to reduce time burdens associated with testing. Federal, state, and local public health entities in Texas each had their own Zika virus–specific testing forms, for which much of the required information overlapped. Condensing these forms into a single, generalizable form could reduce work redundancies and form completion errors while increasing testing efficiency. Ideally, these forms would be online, could translate directly into state databases of testing results, and could be integrated into Zika virus registry reporting information.

### State or Regional Testing Databases

Providing patients with timely testing, clinical management, and follow-up requires that clinicians know of suspected or confirmed exposures. However, our interviews suggested that clinicians faced several barriers accessing patients’ prior Zika virus testing information at the point of care. Insufficient information about prior testing or exposure was particularly challenging for patients who accessed different providers throughout the course of a pregnancy, such as receiving prenatal care from a federally qualified health center, delivering at a county hospital, and going to a separate Medicaid clinic for their neonatal visits. This situation is not uncommon for patients in the demographic group most affected during Houston’s Zika virus experience. For most patients, standard practice for Zika virus testing and reporting of results in Texas occurs through county health departments, according to ZIP code. Such practices are particularly challenging for providers in large medical centers, which regularly draw patients from >1 health department. A potential solution would be to create a centralized state or regional database for Zika virus test results. Centralized databases could enable providers to access timely information about prior exposure and testing history, regardless of where that testing occurred, similar to those that already exist for patients with syphilis or HIV infection.

### Joint Academic–Public Health Task Force

As emerging arboviruses become the new normal, emerging infectious diseases will continue to pose complex health challenges for communities (*17*). Prior responses demonstrate the value of infrastructure and capacity building to prepare for the inevitability of future outbreaks (*18*). Although national organizations and their state and local affiliates (e.g., CDC, US Department of Health and Human Services, American Medical Association, American College of Obstetrics and Gynecology, American Academy of Pediatrics, and Infectious Disease Society of America) can provide information, guidelines, and algorithms, operationalizing and implementing these resources in clinical settings requires local engagement and adaptation. As a public health official explained, “The algorithms… make sense from the scientific viewpoint, but they get confusing to individual practitioners. So making it easy for individual practitioners on the front line to be able to know who to call to get advice… mapping out those systems of care at the local level, I think, will be very important.”

Two levels of local engagement would support the response to emerging infectious diseases: at the community level and within individual institutions. At the community level, task forces composed of health leadership organizations, academic medical centers, and local public health authorities could facilitate communitywide collaboration, addressing such issues as defining roles and responsibilities across partners, communicating to the public about preparedness and response, enhancing laboratory capacity, and expanding testing and surveillance in high-risk areas. This strategy is consistent with prior guidance regarding the importance of training local professionals willing to collaborate with the government in public health management in enabling effective responses to emerging infectious diseases (*19*)*.* Such collaboration will admittedly be challenging in some circumstances because local institutions may be more accustomed to viewing each other as competitors for clinical, philanthropic, and research resources rather than as collaborative partners. Prior successful multiorganizational collaborative efforts may offer some instructive lessons, including enlisting an existing reputable organization as a convening body (e.g., the Texas Medical Center), involving key stakeholders, and using electronic tools for low-cost dissemination (*20*).

At the institutional level (including hospitals, clinics, and academic health centers), leadership should designate an emerging infectious disease point person or committee with the authority and support to rapidly create an ad hoc task force comprising interdisciplinary members needed to effectively address a public health emergency. Diverse representation will be vital to the success of such committees, including clinical leadership to translate national, state, or local algorithms to clinical management plans for the respective institutional contexts; health information management leaders to modify and implement EHR changes to support the clinical response; laboratory personnel to guide testing processes; and communications experts to disseminate guidance on policy and process throughout the institution.

## Limitations

For this project, we purposefully selected clinical, operational, and public health leaders with experience responding to Zika virus infections. Consequently, obstetricians, pediatricians, and infectious disease specialists working in academic medical centers were overrepresented. Their experiences shed insight into features of health system capacity and preparedness to respond to an emerging infectious disease. However, involvement of other stakeholders, particularly patients and primary care providers in nonacademic, rural, and resource-poor settings, probably would have provided additional insights.

The suggestions for improvement reflect issues raised by our respondents and are not exhaustive. Other critical issues merit further attention, including how healthcare providers can ensure that patients feel safe to access needed health services in the face of contemporary government approaches to immigration enforcement (*21*)*.*

## Conclusions

The emergence of Zika virus brought numerous challenges to the health system in Houston. Although the virus itself was relatively new, many of the issues confronted by providers and public health officials in the face of the disease were far from novel. Instead, the issues were often the result of known, predictable, and recurring shortcomings in our healthcare system. The insights of expert stakeholders led us to suggest several strategies for improving the response to Zika virus and other future emerging infectious diseases.

## References

[1] Pan American Health Organization. Zika cases and congenital syndrome associated with Zika virus reported by countries and territories in the Americas, 2015–2017 cumulative cases [cited 2017 Dec 14]. http://www.paho.org/hq/index.php?option=com_docman&task=doc_view&Itemid=270&gid=42362&lang=en

[2] Center for Disease Control and Prevention. Pregnant women with any laboratory evidence of possible Zika virus infection, 2015–2017 [cited 2017 Dec 14]. https://www.cdc.gov/zika/reporting/pregwomen-uscases.html

[3] Center for Disease Control and Prevention. Cumulative Zika virus disease case counts in the United States, 2015–2017 [cited 2017 Dec 14]. https://www.cdc.gov/zika/reporting/case-counts.html

[4] Boeuf P, Drummer HE, Richards JS, Scoullar MJL, Beeson JG. The global threat of Zika virus to pregnancy: epidemiology, clinical perspectives, mechanisms, and impact. BMC Med. 2016;14:112. 10.1186/s12916-016-0660-0 27487767 PMC4973112

[5] Houston Airport System. Media kit fact sheet [cited 2017 Dec 14]. http://www.fly2houston.com/newsroom/media-kit/fact-sheets/

[6] Kraemer MU, Sinka ME, Duda KA, Mylne AQ, Shearer FM, Barker CM, et al. The global distribution of the arbovirus vectors *Aedes aegypti* and *Ae. albopictus.* eLife. 2015;4:e08347. 10.7554/eLife.08347 26126267 PMC4493616

[7] Texas Department of State Health Services. Don’t give Zika a biting chance [cited 2017 Dec 14]. http://www.texaszika.org

[8] Rac M, Eppes C, Dempster C, Ballas J, Davidson C, Aagaard K. Screening for Zika virus in a high-risk non-endemic urban population: patient characteristics and testing outcomes. Obstet Gynecol. 2017;129:35s. 10.1097/01.AOG.0000514332.47783.0f

[9] Eppes C, Rac M, Dunn J, Versalovic J, Murray KO, Suter MA, et al. Testing for Zika virus infection in pregnancy: key concepts to deal with an emerging epidemic. Am J Obstet Gynecol. 2017;216:209–25. 10.1016/j.ajog.2017.01.020 28126366

[10] Russell K, Oliver SE, Lewis L, Barfield WD, Cragan J, Meaney-Delman D, et al.; Contributors. Update: interim guidance for the evaluation and management of infants with possible congenital Zika virus infection—United States, August 2016. MMWR Morb Mortal Wkly Rep. 2016;65:870–8. 10.15585/mmwr.mm6533e2 27559830

[11] Centers for Diease Control and Prevention. CDC updates guidance for pregnant women and women and men of reproductive age for Zika virus infection related to the ongoing investigation of local mosquito-borne Zika virus transmission in Miami-Dade County, Florida [cited 2017 Dec 14]. https://emergency.cdc.gov/han/han00398.asp

[12] Arafat R, Arnold R, Persse D. Preparing for Zika virus in Houston: a comprehensive, city-wide approach. Int J Infect Dis. 2016;53S:72. 10.1016/j.ijid.2016.11.182

[13] Ash JS, Berg M, Coiera E. Some unintended consequences of information technology in health care: the nature of patient care information system-related errors. J Am Med Inform Assoc. 2004;11:104–12. 10.1197/jamia.M1471 14633936 PMC353015

[14] Boonstra A, Broekhuis M. Barriers to the acceptance of electronic medical records by physicians from systematic review to taxonomy and interventions. BMC Health Serv Res. 2010;10:231. 10.1186/1472-6963-10-231 20691097 PMC2924334

[15] Van Beneden CA, Pietz H, Kirkcaldy RD, Koonin LM, Uyeki TM, Oster AM, et al. Early identification and prevention of the spread of Ebola—United States. MMWR Suppl. 2016;65(3):75–84. 27386933 10.15585/mmwr.su6503a11

[16] Wong AE, Garcia PM, Olszewski Y, Statton A, Bryant Borders A, Grobman WA, et al. Perinatal HIV testing and diagnosis in Illinois after implementation of the Perinatal Rapid Testing Initiative. Am J Obstet Gynecol. 2012;207:401.e1–6. 10.1016/j.ajog.2012.08.006 22939690

[17] Hotez PJ, Murray KO. Dengue, West Nile virus, chikungunya, Zika-and now Mayaro? PLoS Negl Trop Dis. 2017;11:e0005462. 10.1371/journal.pntd.0005462 28859086 PMC5578481

[18] Paules CI, Eisinger RW, Marston HD, Fauci AS. What recent history has taught us about responding to emerging infectious disease threats. Ann Intern Med. 2017;167:805–11. 10.7326/M17-2496 29132162

[19] Broome CV. Effective global response to emerging infectious diseases. Emerg Infect Dis. 1998;4:358–9. 10.3201/eid0403.980303 9716944 PMC2640279

[20] Fisher JW, Peek KE. Collaborating for change: creating a women’s health network. Womens Health Issues. 2009;19:3–7. 10.1016/j.whi.2008.10.003 19111782

[21] Saadi A, Ahmed S, Katz MH. Making a case for sanctuary hospitals. JAMA. 2017;318:2079–80. 10.1001/jama.2017.15714 29049516

